# Transnational public and global health education in China

**DOI:** 10.1186/s41256-023-00305-2

**Published:** 2023-06-21

**Authors:** Stephen W. Pan

**Affiliations:** 1 Department of Health and Environmental Sciences, Xi’an Jiaotong-Liverpool University, 111 Ren’ai Road, Suzhou, 215123 Jiangsu China; 2 Department of Public Health, University of Texas at San Antonio, San Antonio, USA

**Keywords:** Joint-venture university, Sino-foreign cooperative university, International health, Public health, Study abroad, Global health workforce

## Abstract

Transnational public and global health programs in China have rapidly expanded over the past 20 years, and have potential to make important contributions to China’s global health workforce. However, there has been sparse if any literature specific to transnational public and global health higher education in China. In response, this perspective article aims to: (1) outline current transnational public and global health programs in China, and (2) delineate opportunities and challenges for transnational public and global health programs to enhance China’s global health workforce. Based on internet searches, eight active transnational public and global health programs in China were identified in September 2022 (one Bachelors; four Masters; three doctorate). Degree awarding institutions are located in Australia, Portugal, the United Kingdom, and the United States. Courses for stand-alone transnational programs were co-delivered by faculty from the Chinese and foreign sponsoring institutions. The earliest and latest programs were respectively established in 2001 and 2022, and the average year of establishment was 2013. The endurance of some programs (three programs operating ≥ 10 years) indicates the potential sustainability of transnational public and global health programs in China. However, opportunities for cross-cultural engagement appear to be constrained by lack of English (or other language) requirements in some programs, limited recruitment of international students, pandemic travel restrictions, and a dearth of funding for global health research outside China. In addition, students enrolled at transnational universities in China are currently ineligible for China Scholarship Council funding. As China’s need for global health capacity grows amid a rapidly shrinking population of younger citizens, strategic investments in transnational public and global health programs may be of increasing value.

## Introduction

Transnational education has been defined as “all types of higher education study programs, or sets of courses of study, or educational services (including those of distance education) in which the learners are located in a country different from the one where the awarding institution is based” [1]. Hence, the primary distinction between transnational education and traditional cross-border education is that the student is not residing in the same country as the degree awarding institution, at least during the time of their study.

Over the past 20 years, transnational public and global health education programs in China have expanded rapidly, driven in part by factors that benefit both individual teachers and students alike. By physically teaching in China for extended periods as “flying faculty” or as permanent employees, transnational education universities and programs offer teachers from degree awarding countries a unique opportunity to glean academic experience in China while maintaining affiliation with the degree-awarding institutions and delivering lectures in English or other non-Chinese languages. This can be especially attractive for faculty interested in conducting research in China but who may not be fluent in the Chinese language.

For students, the allure of transnational education programs in China is driven by desires for international experience, as well as other factors. First, the costs of a transnational education in China will almost invariably be lower than the costs of physically moving and studying in the degree-awarding country of a transnational program (e.g. United States or United Kingdom). Secondly, even if finances are not of great concern, some non-Chinese citizens from the global South may encounter difficulties securing a visa to study in specific industrialized countries. For example, due to geopolitical concerns, students from some countries may face difficulties trying to secure a student visa to the US or other industrialized countries in the global North [2]. Hence, studying at a transnational program in China may be a viable option that enables non-Chinese citizens to obtain a higher education degree from countries that they may not be able to physically enter. Lastly, some Chinese citizens (and perhaps their parents) may prefer transnational education because it enables them to remain in China. Some students may not have any interest in immigrating abroad, while others may not yet feel prepared to live abroad on their own for an extended period of time. Moreover, physically relocating to a foreign country has profound implications for the development of one’s personal/professional network, particularly during college when many important and long-term social connections are formed. If the student is resolute about remaining in their home country, then they may be reluctant to risk sacrificing existing and potential in-country networks by moving abroad.

Transnational public and global health programs in China are well poised to bolster the skillsets of China’s global health workforce. Since at least the 1960s, China has provided important inspiration and support to Global Health initiatives ranging from the Alma-Ata Primary Health Care Initiative to COVID-19 vaccination campaigns [3]. However, recent research has indicated that China’s Global Health Workforce remains relatively underdeveloped, and requires stronger professional and communication skills training [4]. Due partly to such concerns, in 2017, China’s National Health and Family Planning Commission (currently known as the National Health Commission) established a global health talent pool of skilled public health professionals within China who would be able to respond to short and long-term Global Health initiatives [5].

However, despite the growth of transnational public and global health programs in China, and the evolving needs of China’s global health workforce, to date there has been little if any published analysis specific to this issue. In response, this perspective article aims to: (1) outline current transnational public and global health programs in China, and (2) delineate opportunities and challenges for transnational public and global health programs to enhance China’s global health workforce.

### Strengthening China’s global health workforce through transnational global health education

Transnational Global Health Education programs can help strengthen China’s global health talent pool in two key ways. First, university-based transnational global health programs provide a relatively low-stakes setting for prospective global health professionals to enhance their cross-cultural communication and team work skills, given that lectures and curriculum are often delivered in English by international faculty. Opportunities to write reports, deliver presentations, and engage in discussions in English with international interlocutors will invariably help hone non-native English-speaking students’ communication skills that will be applicable to global health projects and engagement with international partners.

Second, transnational global and public health programs can help strengthen professional skills needed for an effective global health workforce. Unlike many public health programs in China which are based within medical schools and have a stronger clinical focus [6], transnational global and public health programs in China tend to provide relatively more interdisciplinary training and program management skills [7, 8]. The following section describes a public health undergraduate course and summer research project which I led at a Sino-British transnational university in China.

### Case example of transnational public health education in China

From 2018 to 2022, I taught an upper-division undergraduate public health course entitled “Methods for analyzing public health: surveillance, monitoring, and evaluation.” The learning objectives of the course focused on enhancing students’ knowledge of public health surveillance and program evaluation, as well as honing students’ research skills. In accordance with Bloom’s taxonomy of learning [9], the learning objectives for this upper division course focused on knowledge creation, as opposed to only knowledge retention and understanding. Students received training on evaluating a public health intervention using an interrupted time-series analysis based on publicly available data. Previous student projects include using UNICEF data to evaluate a breast feeding intervention in Bangladesh and government behavioral surveillance data to evaluate a breast cancer screening intervention in the United States [10]. Assessments were designed sequentially, whereby subsequent assignments build upon previously submitted work, and fellow students and the instructor provide written and face-to-face feedback during class time. Structuring assessments sequentially appears to augment student engagement with instructor feedback [11, 12], and this has been reflected in my own experience.

In the summer of 2020, I conducted a COVID-19 vaccination preferences study with colleagues and undergraduate public health students studying at a transnational university in China. During the data collection phase, COVID-19 vaccines were still in development and there was considerable concern worldwide about vaccine hesitancy and suboptimal vaccine uptake. Hence, the goal of the study was to assess the degree to which vaccine mandates that bar access to public spaces could increase the adult general population’s willingness to vaccinate for COVID-19. To that end, our team conducted a nationwide online discrete choice experiment in China. Undergraduate team members received training in discrete choice experiments and gained considerable experience in project management, cross-cultural team building, and the academic publication process. Findings of the study were eventually published in an international academic journal with an undergraduate public health student as lead author [13]. I believe these learning experiences strengthened students’ professional and cross-cultural communication skills in ways that are applicable to global health programs and projects.

### Current transnational public and global health programs in China

Although transnational universities and programs have expanded rapidly in China over the past 20 years, there has been sparse if any literature that has specifically surveyed transnational public and global health programs. In response, I sought to identify and summarize key features of active transnational public and global health programs in China. Programs were identified by searching for the key word “health” (健康, 卫生) in the Ministry of Education’s “Chinese-Foreign Cooperation in Running Schools” (中外合作办学) database (https://www.crs.jsj.edu.cn/) (date of search: September 23, 2022). Programs deemed to be within the scope of public or global health were included in the analysis, and additional information about each program was gleaned from publicly accessible institutional and program websites.

As of September 2022, there were eight active transnational public and global health programs that have been approved by the Ministry of Education of the People’s Republic of China (Table 1). Foreign degree awarding institutions are located in Australia, Portugal, United Kingdom, and the United States. Chinese partner institutions are located throughout China, ranging from Haikou in the South to Harbin in the North. Approved programs include one Bachelor program, four Masters programs, and three doctoral programs. The earliest and latest programs were respectively established in 2001 and 2022, and 2013 was the average year of establishment. Of the eight programs, four are delivered at a transnational university or college, and four are delivered at the Chinese university. Target student enrollment for most Masters programs was 80 students per year, and one doctoral program aims to enroll 25 students per year. Another doctoral program established in 2015 has ceased enrollment of new students. No target enrollment information was found for the Bachelor program.

**Table 1:** Transnational Public and Global Health programs in China (September 2022)

Institutions	Year estab-lished	Level of transnational cooperation	Target enrollment program (s)^1^	Language of application website	English proficiency criteria required for admission?	Delivery of courses	Transnational public health programs^1^
Benedictine University (BU)—Dalian Medical University (DMU)^2^	2012	Program	80 students	Chinese^2^	None found	Co-taught by BU and DMU faculty; English lectures translated into Chinese	Master of Public Health (Benedictine University, USA)
Duke-Kunshan University (DKU)^3^	2014;2017	University	Not reported	English^3^	Yes	All courses taught in English by DKU and Duke faculty	Master of Science in Global Health; Bachelor of Science (Global Health concentration) (Duke University, USA)
Harbin Medical College (HMC)—Latrobe University (LU)^4^	2001	Program	80 students	Chinese^4^	No	Co-taught by HMC and LU faculty; English lectures translated into Chinese	Master of Health Administration (La Trobe University, Australia)
ISCTE Lisbon University Institute, Portugal (ISCTE-IUL)—Southern Medical University (SMU)^5^	2012	Program	25 students per year	Chinese^5^	No	Co-taught by ISCTE-IUL and SMU faculty; English lectures translated into Chinese	Doctor of Management (Public Health Policy and Management concentration) (ISCTE Lisbon University Institute, Portugal)
Tsinghua University—Johns Hopkins University (JHU)^6^	2015	Program	No plans for future enrollment^6^	English^6^	Yes	Courses delivered in China and USA by JHU-affiliated faculty*	Doctor of Public Health (Johns Hopkins University, USA)
UWE College of Hainan Medical University (HMU)^7^	2022	College	80 students per year	Chinese^7^	Yes. English language prep program available if proficiency is insufficient	Co-taught by UWE and HMU faculty*	Master of Science in Public Health (University of the West of England, UK)
Xi’an Jiaotong-Liverpool University (XJTLU)^8^	2014	University	Not reported	English^8^	Yes	All courses taught in English by XJTLU and UoL faculty	Doctor of Philosophy in Public Health (University of Liverpool, UK)

^*^Language of instruction not found

^1^https://www.crs.jsj.edu.cn/; ^2^https://mph.dmu.edu.cn/xxjj.htm; https://ben.edu/academics/asia-programs/; ^3^https://globalhealth.dukekunshan.edu.cn; ^4^https://www.hrbmu.edu.cn/info/1044/3660.htm; ^5^http://portal.smu.edu.cn/wsglxy/info/1219/2925.htm; ^6^https://publichealth.jhu.edu/academics/jhu-tsinghua-doctor-of-public-health; ^7^https://www.hainmc.edu.cn/hmu_uwe/xygk/gywm1.htm; ^8^https://www.xjtlu.edu.cn/en/study/phd/public-health

Four program application websites were only available in Chinese, thus suggesting that these programs were focused on recruiting students domestically from within China. English language proficiency criteria are not required for admission to at least three of the stand-alone transnational programs. Programs delivered at transnational universities are exclusively taught in English by institutional faculty (e.g., Duke Kunshan University), but at least three stand-alone transnational programs are taught in both Chinese and English by faculty from the Chinese and foreign institutions (e.g., Dalian Medical University – Benedictine University joint Master of Public Health program). English to Chinese interpretation/translation is available in stand-alone programs which have no English language proficiency requirements for admission. One transnational Master’s program provides a 5–15 week English prep program for prospective students who are unable to meet the minimum English language requirements for admission.

### The future of transnational public and global health programs in China

Transnational public and global health programs have strong potential to enhance critical communication and professional skills among China’s developing global health workforce. However, tapping into this potential will require strategic planning and deliberate effort. Currently, several established transnational programs have little to no English language requirements for admission. Foregoing such language requirements clearly widens the pool of eligible program applicants from China, but is not without opportunity costs. First, Chinese students may lack meaningful opportunities to hone their English language communication and teamwork skills if the entire program can be completed in Chinese. For Chinese students who have already entered the domestic workforce, the transnational program may represent one of the few opportunities to experience extended professional and academic engagement with foreign colleagues. It is reasonable to expect that as students’ English language communication skills sharpen, so too will their ability to effectively contribute to international global health initiatives. For students who are interested in transnational programs but are unable to meet the minimum English language requirements for admission, program administrators can consider offering intensive English language prep programs prior to enrollment in subject matter courses, as one transnational college in Hainan province is currently doing.

Second, tailoring program recruitment and curricula for Chinese speaking students excludes the large population of potential international students who are not fluent in Chinese. International students can enrich the learning experience by increasing student opportunities to strengthen cross-cultural communication, teamwork, and professional networks that will endure well beyond graduation. These prospective international students may also become of increasing interest to higher education program directors in general as China’s population rapidly ages and the cohort of young adults shrinks. In 2012, when two transnational public health programs were established, there were approximately 119.6 million Chinese residents between the ages of 20–24 [14]. However, this population has dropped to ~ 80.1 million as of 2022, and is projected to decline to ~ 66.5 million by 2042 [14]. That said, the effect of demographics on student enrollment numbers may be partially mitigated by broader global trends. Travel restrictions, anti-Asian sentiments [15], and geopolitical tensions appear to have dissuaded some Chinese students from studying overseas [16]. If such trends intensify, then enrollment of Chinese students into transnational programs may be adequate as more Chinese students opt to obtain foreign educational credentials from within China rather than abroad. Of course, the sustainability of transnational programs rests upon not just economic viability, but sufficient stakeholder consensus and geopolitical stability as well [17]. It would be prudent for transnational program administrators to continue examining the extent to which current enrollment strategies focused on Chinese citizens are sustainable in the face of medium to long-term demographic and geopolitical trends.

Along that vein, it is important to remain clear-eyed about the inherent challenges of transnational public and global health programs. First, limited English proficiency may complicate comprehension of some lectures and reading material, especially during the first year of the program. Students who are not native English speakers may find it more difficult to improve their English language skills given that socializing outside of school may not require the use of English. In an official national survey of transnational education universities and programs in China, students’ English proficiency level was the most commonly cited problem by faculty and staff at transnational institutions of higher education [18].

Second, maintaining the quality of the awarding institution in a foreign country can be daunting at times. To begin with, it may be difficult to recruit high quality faculty to the transnational education program. Qualified faculty members may be unable or unwilling to live in the host country for extended periods of time, thus significantly reducing the pool of applicants from qualified faculty. One proposed solution has been to use “flying faculty,” whereby faculty permanently posted at the degree awarding institution fly into the host country to deliver several weeks of intense classes. However, compressing a semester’s worth of material into several weeks can be taxing on both student and instructor, and may not allow the students enough time to thoroughly digest the material and develop well-thought written assessments [18]. Moreover, travel restrictions imposed during the COVID-19 pandemic acutely highlighted the risks of flying faculty and other educational arrangements heavily dependent on unimpeded international travel [19].

Third, conflicting policies and cultural norms can also engender tensions that could potentially compromise the learning experience of students. For example, numerous learning resources may be located on websites (e.g., www.youtube.com) that cannot be accessed due to local government internet restrictions [20]. Hence, students at transnational universities may potentially face more obstacles to accessing learning materials, compared to students studying at the degree awarding institution’s home campus. In addition, dual, parallel degrees (one from the host country and one from the degree-awarding country) common in transnational education programs can become complicated by different assessment standards. For example, in the UK, marks as low as 40 are deemed passing, and faculty often use the full range of the marking scale. In contrast, in traditional Chinese universities, marks below 60 are considered as failing, and faculty typical cluster marks of students more closely together [21]. Therefore, when marks for a single course are applied to dual degree programs, there may be a lack of consensus for which marking standard should prevail.

Fourth, transnational universities and programs currently encounter some unique funding challenges. For example, the national government’s China Scholarship Council enables many students from the global South to study at traditional Chinese universities and programs, but transnational universities and programs have been ineligible for such funding. Thus, transnational universities and programs must be proactive about funding student scholarships through alternative means. Extramural research grants can be a potentially important source of revenue, but international faculty with limited Chinese proficiency may find it challenging to develop compelling research grants in Chinese. International faculty employed at transnational universities can submit English language research proposals to funding programs specifically designed for foreign researchers (e.g., the Research Fund for International Scientists), but global health research funding often cannot be spent outside China [3]. Under the auspices of South-South programs such as the Belt and Road Initiative, China’s government can develop global health funding streams explicitly focused on international projects and allow prospective applicants to apply in either English or Chinese. Mitigating language barriers for global health funding opportunities will likely enable a greater pool of international global health researchers at transnational universities (as well as traditional domestic universities) to help build China’s global health workforce and spur greater opportunities for student engagement in global health.

## Conclusion

Transnational public and global health programs in China have strong potential to enhance cross-cultural communication and professional skills of China’s global health workforce. However, opportunities for cross-cultural engagement are constrained by lack of English (or other language) requirements, limited recruitment of international students, pandemic travel restrictions, and a dearth of funding for global health research outside China. As China’s need for global health capacity grows amid a rapidly shrinking population of younger citizens, strategic investments in transnational public and global health programs may be of increasing value.

## Data Availability

Not applicable.

## Abbreviations

COVID-19: Coronavirus disease 2019
BU: Benedictine University
DKU: Duke-Kunshan University
HMC: Harbin Medical College
LU: Latrobe University
UoL: University of Liverpool
UWE: University of the West of England
DMU: Dalian Medical University
ISCTE-IUL: ISCTE Lisbon University Institute, Portugal
SMU: Southern Medical University
JHU: Johns Hopkins University
HMU: Hainan Medical University
XJTLU: Xi'an Jiaotong-Liverpool University
UK: United Kingdom
UNICEF: United Nations Children's Fund
US: United States

## References

[1] Council of Europe. Code of good practice in the provision of transnational education. 2002.

[2] Altbach PG, Knight J (2007). The internationalization of higher education: motivations and realities. J Stud Int Educ.

[3] Xu D, Cheng F, Chen Y, Hao Y, Wasserheit J, China University Global Health Writing Group. Harnessing China’s universities for global health. Lancet. 2016;388:1860–2. 10.1016/S0140-6736(16)31839-6 27751382

[4] Ma X, Ding W, Qian Y et al. Deployment of workforce in global health: what should be the priorities for China?. Glob Health Res Policy. 2021;6:22. 10.1186/s41256-021-00208-0 10.1186/s41256-021-00208-0 PMC8258270 34229758

[5] Fu J, Jiang C, Wang J, Xing Y (2018). To establish a talent pool for global health in China: from political will to action. Glob Health Action.

[6] Jin H, Dong G, Zou L, Shen X, Li D (2020). History and status quo of higher public health education in China. Public Health Rev.

[7] Duke Kunshan University. Global Health with Tracks in Biology and Public Policy. 2022; 1–9. https://ugstudies.dukekunshan.edu.cn/majors/global-health-with-tracks-in-biology-and-public-policy/. Accessed 26 Jun 2022.

[8] Leggat SG, Liu C, Wu Q (2018). Sino-Australian University partnership in health management education. Front Public Health.

[9] Krathwohl DR (2002). A revision of Bloom’s taxonomy: an overview. Theory Pract.

[10] He S, Pan SW (2020). Breast cancer screening trends among lower income women of New York: a time-series evaluation of a population-based intervention. Eur J Breast Health.

[11] Ajjawi R, Kent F, Broadbent J, Tai JH, Boud D, Ajjawi R, et al. Feedback that works: a realist review of feedback interventions for written tasks. Stud High Edu. 2022;47(7):1343–56. 10.1080/03075079.2021.1894115

[12] Winstone NE, Boud D (2020). The need to disentangle assessment and feedback in higher education education. Stud High Educ.

[13] Wang J, Wagner AL, Chen Y, Jaime E, Hu X, Wu S (2022). Would COVID-19 vaccination willingness increase if mobile technologies prohibit unvaccinated individuals from public spaces ? A nationwide discrete choice experiment from China. Vaccine.

[14] Department of Economic and Social Affairs - Population Division. World Population Prospects 2022. https://population.un.org/wpp/Download/Standard/Population/. Accessed 29 Sep 2022.

[15] Pan SW, Shen GC, Liu C, Hsi JH, Pan SW, Shen GC (2021). Coronavirus stigmatization and psychological distress among Asians in the United States. Ethn Health.

[16] Zhu J, Ni D. Is Studying Overseas Losing Its Allure for Chinese Students? Sixth Tone. 2022. https://www.sixthtone.com/news/1010364/is-studying-overseas-losing-its-allure-for-chinese-students%3F. Accessed 30 Sep 2022.

[17] Union U and C. Empire built on sand: UCLan ’ s great overseas gamble. UCU Briefing. 2014;1–5. https://www.ucu.org.uk/media/6156/Briefing-Empire-built-on-sand—UCLans-great-overseas-ganble-Feb-14/pdf/UCLan_briefing_feb14.pdf. Accessed 30 Sep 2022.

[18] Hu M, Eisenchlas SA, Trevaskes S (2019). Factors affecting the quality of transnational higher education in China : a qualitative content analysis on Chinese host universities ’ self-appraisal reports. J High Educ Policy Manag.

[19] The Quality Assurance Agency for Higher Education. Effective Practice in UK Transnational Education during the COVID-19 Pandemic. 2020;1–8. https://www.qaa.ac.uk/docs/qaa/guidance/effective-practice-uk-tne-covid-19.pdf?sfvrsn=b0bccc81_4. Accessed 3 Feb 2021.

[20] Chu C (2017). Censorship or protectionism? Reassessing China’s regulation of internet industry. Int J Soc Sci Humanit.

[21] The Quality Assurance Agency for Higher Education. Review of UK transnational education in China 2012 : Overview. 2013; May. https://www.qaa.ac.uk/docs/qaa/international/tne-china-overview-(1).pdf?sfvrsn=e43ff481_2. Accessed 4 Feb 2021.

